# Determinants of Male Partner Involvement in Promoting Deliveries by Skilled Attendants in Busia, Kenya

**DOI:** 10.5539/gjhs.v4n2p60

**Published:** 2012-03-01

**Authors:** Mildred Nanjala, David Wamalwa

**Affiliations:** African Medical and Research Foundation, HIV/AIDS Programme P.O. Box 30125-00100, Nairobi, Kenya Tel: 254-729-509-455 E-mail: mildrednanjala@gmail.com; African Medical and Research Foundation, Child and Reproductive Health Programme P.O. Box 30125-00100, Nairobi, Kenya Tel: 254-733-229-992 E-mail: david_wamalwa@yahoo.com

**Keywords:** Skilled attendant, Male partner involvement, Skilled delivery, Traditional birth attendant

## Abstract

A cross-sectional study covering 380 male partners and their spouses was conducted in Busia district in Western Kenya to establish demographic, socio-economic and cultural factors that affect male partner participation in promoting deliveries by skilled attendants. The study showed a significant relationship between level of education (*P*=0.0000) and level of income (*P*=0.0004) of the male partner and his support for skilled delivery. Lack of knowledge by male partners of complications associated with delivery, cultural beliefs, high fees charged for deliveries at health facilities and “un-cooperative” health workers are major contributing factors to low male partner involvement in child birth activities. Improving the levels of education and income of male partners, addressing the cultural beliefs and practices, improving health care provider-client relationship and sensitizing men on complications associated with pregnancy and child birth can contribute significantly in enhancing male partner involvement in promoting deliveries by skilled attendants.

## 1. Introduction

An estimated 358 000 maternal deaths occurred worldwide in 2008, with the developing countries accounting for 99% of the deaths (WHO, 2010). Improving maternal health is one of the eight Millennium Development Goals (MDGs) adopted by the international community at the UN Millennium Summit in 2000. MDG 5 aims at reducing the maternal mortality ratio (MMR) by 75% between 1990 and 2015, that is; it seeks to achieve a 5.5% annual decline in MMR from 1990. Globally the annual percentage decline in MMR between 1990 and 2008 was only 2.3% (WHO, 2010). In Busia district in Western Kenya where this study was conducted, the maternal and child health care indicators as shown in table 1 are poor compared to the national indicators except for the neonatal mortality rate.

**Table 1:** Maternal and Child Health indicators in the study site (Busia) in comparison to national indicators

Maternal and Child Health Indicators	Busia District (KDHS 2008)	Kenya (KDHS 2008)
Maternal Mortality Ratio	680/100 000	488/100 000
Infant Mortality rate	65/1 000	52/1 000
Neonatal Mortality rate	25/1 000	31/1 000
Deliveries at health facilities	25.3%	43.6%

The African Medical and Research Foundation (AMREF) in collaboration with the Ministry of Public Health and Sanitation (MoPHS) with funding from USAID have since October 2005 been implementing a child survival project to improve the maternal child health indicators in Busia. Whereas the project has achieved significant improvements in a number of maternal and child health indicators, deliveries by skilled providers is still very low, and this is partly attributed to low male partner involvement in child birth issues and specifically failure by them (male partners) in supporting their spouses to access delivery services from skilled attendants (AMREF, 2008). In recent years the role that men play in reproductive health and maternal and child health issues has drawn increased interest. To date, most of these efforts have centred on sexual and reproductive health issues such as condom use, multiple sex partners and decision making about family planning (Drennan, 1998) and (EngenderHealth, 1998). This study sought to establish factors that affect male partner involvement in supporting their spouses to access delivery services from skilled attendants in Busia district, in Western Kenya.

## 2. Research Methods

### 2.1 Study Area

This study was undertaken in Busia district located in western Kenya. The district has five administrative divisions with an estimated population of 415 000. The study catchment area (Butula and Funyula) has an estimated population of 50 000 women of reproductive age and 38 000 children aged less than five years. According to the records kept by the Busia District Medical Officer of Health, the study area at the time of the study had a total of 49 858 households with children aged 0-12 months, and the rate of women accessing deliveries from skilled attendants was 25.5% compared to the national rate of 46.6% (KDHS, 2008).

### 2.2 Study Design, Sampling and Participants

The study was undertaken in November 2009 and employed a cross-sectional study design. The study area was Busia district as the Ministry of Public Health and Sanitation (MoPHS) in collaboration with the African Medical and Research Foundation (AMREF) had been implementing a Child Survival project in area since October 2005. Through the project, it was noted that despite the interventions put in place, the rate of deliveries conducted by skilled attendants was low, and during the mid- term evaluation conducted in October 2008, low male participation in the project activities was identified as partly contributing to this low rate. The study, therefore, sought to establish factors contributing to low male partner participation in supporting their spouses to access delivery services from skilled attendants.

A sample size of 380 male partners and their spouses who at the time of the study had children aged 0-12 months was calculated using a formula by Fishers n=Z^2^Pq/d^2^ (Mugenda and Mugenda, 2003), where n was the desired sample size of the study population, Z was the standard normal deviate, set at (1.96) which corresponds to the 95% confidence level, P was the proportion of male partners in the target population estimated to be supporting their spouses to access delivery services from skilled attendants which was unknown and hence set at 50% (0.5), and d was the degree of accuracy set at 0.05 and q was set at 0.5 (1-0.5).

The period of 0-12 months was thought to be a reasonable time to allow the respondents to recall the activities they carried out during the period of delivery. The male partners and spouses were selected through a simple random technique using a list of households (sampling frame of estimated 49 858 households) with children aged 0-12 months that had been developed by the MoPHS. To validate the information provided by male partners, focus group discussions and key informant interviews were held with community health workers and the in-charges of the maternity wards in the study area.

### 2.3 Data Collection

Both qualitative and quantitative were used in this study. The quantitative data was obtained from male partners and their spouses using structured, interviewer administered questionnaires, while qualitative data was collected from the community health workers using focus group discussion guides, and from in-charges of maternity wards working in health facilities that are utilized by women in the study area using key informant interview guides. Data was collected by enumerators who underwent a one day training which covered three major areas namely; familiarization with the research tools, communication skills, interviewing skills, and ethics in research. Pre-testing of data collection tools was done in Matayos Division which is adjacent to both Butula and Funyula Divisions. One focus group discussion was held with ten community health workers, one key informant interview was held with the in charge of Matayos Health Centre and twenty households were sampled where the male partners and their spouses were interviewed.

### 2.4 Ethical Issues

The research protocol was submitted and approved by the Ethics and Scientific Research Committee of Great Lakes University of Kisumu (GLUK). Before obtaining the participants consent, the researcher explained to them the purpose of the interview, the content of the interview, and the confidentiality of the recorded information. In addition, they were informed that they could quit the interview at any stage. A written informed consent was obtained from the participant, and the interview was recorded after obtaining the written consent.

### 2.5 Data Analysis

The socio-demographic and economic variables namely male partner’s age, educational level, occupation and cultural beliefs, as well as the level of knowledge regarding complications arising from child-birth was sought to establish if it could affect male’s participation in supporting his spouse in accessing skilled delivery services. From the spouses, the study sought to establish the role that the male partners played if any in enabling them access delivery services from skilled attendants. Quantitative data was analysed using computer statistical software of Statistical Package of Social Sciences (SPSS) version 10.1 while the qualitative data was analysed using content analysis. Statistical analysis using the odds ratio was done to test the association between the occupation, level of education and age of male partner and his involvement in supporting his spouse to access skilled delivery services. Odds ratio was also done to test the association between the age of the spouse and her utilization of skilled attendant for delivery services.

### 2.6 Analysis of Results

About 44% of the deliveries in the study area are conducted by traditional birth attendants compared to 28% in Kenya, and the percentage of mothers delivering at health facilities in the study is only 31.5% compared to the national percentage of 43% as shown in table 2.

**Table 2:** Types of delivery services as established by research findings in comparison to the KDHS 2008 findings

Type of delivery service	Research Findings	KDHS 2008 Findings
Deliveries by skilled providers	32.9%	44.0%
Deliveries by Traditional Birth Attendants	44.0%	28.0%
Deliveries at health facilities	31.5%	43.0%

The study established no statistical significance between the male partner’s age and support for skilled delivery services for the spouses (p=0.4259). The study, however, established that the male partner’s level of education (P=0.0000) and occupation (P=0.0004) have an effect on his involvement in supporting his spouse to access delivery services from skilled attendants. A statistical analysis using odds ratio was also computed to establish the association between the spouses’ age and access to delivery services from skilled attendants and this was statistically significant (P=0.000).

The study established that socio-cultural factors affect the involvement of male partners in supporting their spouses to access skilled delivery services. For instance, about 33% of the male partners stated that their newly born babies must be kept indoors for three days for boys and two days for girls and taken out only after the naming ceremony and about 45% of them said that child-birth is a woman’s affair which does not require their participation, 39% stated that the placentas must be buried secretly to avoid babies from being bewitched which they felt was not possible if deliveries took place at health facilities. Most of the male partners also have limited knowledge regarding complications related to child-birth as 40% stated that delivery is a natural phenomenon that does not require men’s participation, and 48.2% of the male partners said that they will be ridiculed by their peers and be seen as being “ruled” by their wives if they were seen accompanying them to health facilities for delivery.

**Table 3:** Knowledge and cultural factors affecting male partner involvement in promoting skilled deliveries

Cultural/Knowledge factors	N=380	%
Babies should be kept in-doors till naming ceremony	25	32.9
Child-birth is a woman’s affair that does not require men participation	170	44.7
Placenta must be disposed secretly which is not possible with hospital delivery	147	38.7
Child-birth is natural phenomenon that should not be given much attention	153	40.3
Accompanying wife to hospital for delivery will result to the man being ridiculed	83	48.2

Majority of the male partners (61.1%) stated that the fees charged at health facilities was high and beyond their reach, 54% indicated that the health workers are harsh and uncooperative, an assertion that was supported by community health workers (CHWs) during focus group discussions. One CHW remarked *“we make efforts in persuading the male partners to accompany their spouses to health facilities but our efforts are frustrated as they (men) are abused by the health workers and asked if they have also gone to hospital to deliver”*. Also about 48% of the male partners stated that when they accompany their wives to the antenatal clinics, they are forced to take HIV tests without adequate counseling. In spite of lack of support by male partners to the spouses, most mothers (72%) interviewed felt that their male spouses should at least set aside funds to be used in assisting them access skilled delivery services while 54% indicated that they wanted their male partners to be accompanying them to health facilities for antenatal care and delivery services.

**Table 4:** Factors affecting male partners from supporting their spouses to access delivery services at health facilities and expectations of females from their male spouses

Why male partners do not support their spouses to access hospital deliveries	N=380	%
Health workers are uncooperative and harsh	208	54.7
Forced to undertake a mandatory HIV test	184	48.4
High fees charged for delivery services	232	61.1

**Type of support expected by female from their male partners**		

Be accompanied by their male spouses to health facilities	204	53.7
Male partners to set aside a budget for delivery services	290	71.5

**Table 5:** The association between the male partner’s age and the spouse’s access to delivery services from skilled attendant

Male partner’s age in completed years	Spouse delivered by a skilled attendant				

	Yes	No	P. value	OR at 95% level of confidence	Confidence interval
Aged 29 years or below (N=179)	63	116	0.4259	1.190	0.758-1.867

Aged 30 years or above (N=201)	63	138

**Table 6:** The association between the male partner’s occupation and the spouse’s access to delivery services from skilled attendant

Male partner’s occupation	Spouse delivered by a skilled attendant				

	Yes	No	P. value	OR at 95% level of confidence	Confidence interval
Unemployed (N=191))	47	144	0.0004	0.455	0.286-0.720

Employed or running a business (N=189)	79	110

**Table 7:** The association between the male partner’s level of education and the spouse’s access to delivery services from skilled attendant

Male partner’s level of education	Spouse delivered by a skilled attendant				

	Yes	No	P. value	OR at 95% level of confidence	Confidence interval
Primary level of education and below (N=243)	63	180	0.0000	0.235	0.147-0.375

Secondary level of education and above (N=137)	84	53

**Table 8:** The association between the mother’s age and access to delivery services from skilled attendant

Age of the mother in completed years	Delivered by a skilled attendant				

	Yes	No	P. value	OR at 95% level of confidence	Confidence interval
≤ 35 years (N=221)	105	116	0.0000	10.166	5.235-20.628

≥ 36 years (N=159)	13	146

## 3. Discussion

### 3.1 Age of Male Partner and Spouse

The study established no significant statistical difference between the age of a male partner and the type of delivery (skilled or unskilled) of the spouse (P=0.4259). This study finding agrees with the findings of a study conducted by Babalola and Adesegun (2009) which established no significant statistical relationship between age and use of skilled assistance during delivery. The analysis of the study findings established that the number of women who were attended to by skilled attendants during birth was seen to decrease with increasing age so that young women of aged 35 years and below were more likely to seek services from skilled attendants than women aged 36 years and above (P=0.0000). This study finding is similar to the survey conducted by Family Care International (2001) which established that young women were more likely to seek skilled care at delivery because they fear the risks involved in pregnancy and also because majority are first time mothers. The finding also shows similarity to that of a cross sectional study done in 2007 in Southern Tanzania by Mpembeni *et al*, which established that younger women are more likely to seek skilled care at delivery. Mpembeni *et al* noted that “younger women are just starting child bearing and are told to be in a high risk group and so they tend to fear home deliveries”.

### 3.2 Education Level of Male Partner

The study findings show a significant statistical difference between the educational level of the male partner and the type and place of delivery (P=0.0000). More spouses of male partners with secondary level of education and above sought skilled care at delivery than the spouses of less educated male partners. It can be argued that male partners with some basic level of education better understand the complications associated with unskilled delivery. Education also enables men to discard the negative attitudes and cultural beliefs, and it is also likely that men with high level of education have some formal employment which enables them to raise funds that they can use to pay hospital bills for delivery services. Similar findings were established in a study of Salvadoran fathers’ by Carter and Speizer (2005) which showed that attendance at prenatal care, delivery and postpartum care where men with more than a primary school education were more likely than their less educated counterparts to participate in birth related activities. The Salvadoran study further established that educated mothers were more likely to seek skilled care due to increased knowledge of the benefits of preventive health care and awareness of health services, higher receptivity to new health-related information, socialization to interact with formal services outside the home environment, familiarity with modern medical culture, access to financial resources and health insurance, more control over resources within the household and wiser spending, more egalitarian relationship and better communication with the husband, more decision-making power, increased self-worth and self-confidence, better coping abilities and negotiating skills as well as reduced power differential towards health care providers and thus better communication and ability to demand adequate services. The study findings also agree with the findings of a study conducted by Iliyasu *et al* (2010) in Northern Nigeria to assess birth preparedness, complication readiness and father’s participation in maternity care, which established that husbands with formal education were more likely to participate in maternity care compared to those with non-formal education.

### 3.3 Effects of the Male Partner’s Occupation on the Type of Delivery Accessed by Spouse

The study established that spouses of male partners with formal employment or engaged in a businesses were more likely to seek skilled delivery than those whose partners were unemployed. An odds ratio to assess if a relationship between the occupation of a male partner and the type of delivery the spouse seeks exists showed a significant statistical difference (p=0.0004). A chi-square tabulation also established a significant statistical difference between the mother’s occupation and the type of delivery that she accesses (p=0.02). These findings clearly show that spouses of male partners with a high level of income are more likely to be delivered by skilled attendants than those whose partners have a low level of income. This is because male partners with incomes are able to pay the delivery fees at health facilities for their spouses. Similar findings were reported by a cross sectional study done in Southern Tanzania by Mpembeni *et al* (2007) whose study established that women of high economic status sought skilled care at birth because they were able to make wise decisions about their health and meet the costs of the same. A cross sectional study conducted in Kadune State in Nigeria Idris *et al*. (2006) also established that employment status of husbands was an important determinant of the place of delivery as wives of employed husbands delivered at the hospital.

### 3.4 Knowledge

Majority of male partners exhibited very low knowledge regarding complications that are associated with pregnancy and delivery, with 24.9% of them responding that they did not know a single complication and those who were able to mention at least one complication were very few. Majority of the mothers and focus group discussion discussants also reported that men regarded delivery as a natural phenomenon and hence saw no need of being involved. It can be argued that the thinking of men that delivery is a natural phenomena arise from their lack of knowledge regarding the complications that are associated with pregnancy, labour and delivery, and that perception has affected the male partner involvement in supporting their spouses in accessing delivery services from skilled attendants. These findings agree with those of Mullany *et al* (2006) who, in a randomised controlled trial conducted in urban Nepal established that educating mothers and spouses to understand the complications of pregnancy and child birth led to an increased uptake of maternal and child health services.

### 3.5 Effects of Male Partner’s Cultural Beliefs on the Type of Delivery Service by Accessed Spouse

From the study findings, cultural beliefs of male partners contribute to non-participation of men in pregnancy and child birth. The focus group discussions with the community health workers established that male partners do not get involved in child birth and delivery because clinic issues are perceived by men as a woman’s affair. These findings agree with a study done by JHPIEGO (2001) that pregnancy and child birth continue to be viewed solely as a woman’s affair Some men reported that they would be regarded by relatives and fellow male colleagues as being “ruled” by their wives if they were seen taking part in child birth issues. Men also insist that their wives should deliver at home because they believe newly born children are supposed to stay indoors for up to four days for the boy child and three days for the girl child until when they are named to avoid bad omen. They further say that the deliveries should take place at home to ensure that the placenta is safely buried to avoid someone stealing it and bewitching the baby. These negative cultural beliefs prevent the parents from seeking skilled maternity care during delivery. The study also established that male partners value being respected and will seek for services from people that are “polite to them and provide nice care”. This response was given by majority of the male respondents when asked why they preferred traditional birth attendants’ (TBAs) services as opposed to those of health workers at health facilities. Majority of the men said that when they accompany their spouses to health facilities they are always “ridiculed” and not treated as “men”. This pronouncement by men clearly shows that they are still embedded in some cultural practices that are negatively impacting on seeking modern health care services including skilled deliveries for their spouses The study findings agree with those of a study that was done in Nigeria by Wall (1998) to establish the social context of maternal morbidity and mortality among the Hausa of Northern Nigeria which established a strong influence of cultural beliefs and practices on childbirth and fertility-related behaviors. Also, a study conducted in South Africa by Saiqa *et al* in 2000 established similar findings with male respondents saying that “it is unheard of for men to be involved in the care of their infant children. They are not permitted to see the mother or child for three months after birth as doing so will make the males “weak”.

## 4. Conclusions and Recommendations

Low levels of education and income, negative cultural practices of male partners, negative attitudes of health workers, unavailability of skilled attendants at community level, the “nice care” offered by the traditional birth attendants, and “high fees” charged at health facilities for delivery services have affected the participation of male partners in promoting skilled deliveries for their spouses. Majority of the male partners exhibited negative views towards issues related to pregnancy and child birth with most of them saying that delivery is a “woman’s affair” and a natural phenomena and therefore they see no need of being involved. A very high percentage of male partners indicated that they would be ridiculed by their peers and relatives if they were seen participating in pregnancy and child birth issues. This negative attitude is one of the contributing factors to the low rate of male partner involvement in child birth issues.

Improving the levels of education and income of male partners, addressing the cultural beliefs and practices, improving health care provider-client relationship and sensitizing male partners on complications associated with pregnancy and child birth can go a long way in promoting male partner involvement in promoting deliveries by skilled attendants.

## Appendix:

Key research findings presented in tables
